# P01-003 – Bleeding disorder in FMF

**DOI:** 10.1186/1546-0096-11-S1-A7

**Published:** 2013-11-08

**Authors:** O Karadag, T Akin Telli, N Sayinalp, I Ertenli

**Affiliations:** 1 Division of Rheumatology, Ankara, Turkey; 2 Division of Haematology, Ankara, Turkey, Hacettepe University Faculty of Medicine, Ankara, Turkey

## Introduction

The most serious complication in Familial Mediterranean fever (FMF) is the development of amyloidosis, which usually determines the prognosis. Amyloid deposition can be systemic or organ-specific. The clinical features of amyloidosis are dependent on the organs involved, type of amyloidosis, *rate* of *amyloid deposition and amount of amyloid fibrils. Organ dysfunction can cause life*-*threatening bleeding.* Amyloid deposition is the main cause of abnormal bleeding but also coagulation factor deficiencies, hyperfibrinolysis, platelet dysfunction and amyloid angiopathy with increased fragility of blood vessels can be regarded as other important pathogenetic factors. Herein a case of FMF amyloidosis with splenomegaly, refractory cytopenia and bleeding disorder is presented.

## Case report

Thirty-one year old male patient was admitted with complaints of upper gastrointestinal (GI) bleeding and hematuria.*In his past medical history*, *he had recurrent attacks of abdominal pain and fever since early childhood and 9 years ago a renal biopsy* was *performed* to evaluate proteinuria and AA amyloidosis was detected. He had been diagnosed as FMF and colchicine treatment was begun. On physical examination, *ecimotic* skin *lesions* on lumbar area and legs were found. The abdominal examination revealed painless massive splenomegaly palpable up to the pelvis. Also physical examination revealed markedly thickened skin in each ear in the area of concha and amyloid nodules of ear. Laboratory results were as follows: Hemoglobin level 8.8 g/dL, white blood cell count:9000/ mm3, platelet count 41000/ mm3 , INR level 1.7. Peripheral blood smear examination showed *poikilocytosis*, *acanthocytosis*, neutrophils %61, *lymphocytes* %38 , platelet count compatible with thrombocytopenia. LDH level was normal and coombs tests were negative. *Beta*-*2 microglobulin level was too high.*( 13417 ng/ml). Bone marrow aspiration and biopsy revealed normocellularity of the marrow and *deposition* of *amyloid in* the walls of the blood *vessels. All coagulation factor levels* were decreased in plasma. *As the reason of bicytopenia peripheral degradation and splenic sequestration were evaluated primarily. Amyloidosis caused platelet aggregation defect. Because of the high bleeding risk, he* underwent radiotherapy *instead of splenectomy for hypersplenism. Despite* splenic irradiation *thrombocytopenia didn’t improve, radiation therapy didn’t shrink* the spleen. Genetic mutation analysis showed homozygous M694V alleles on MEFV gene. Red blood cells, platelets, fresh frozen plasma and fibrinogen were transfused in order to improve *bleeding diathesis. On follow-up* the nosocomial infections led to exitus through a septic shock.

## Discussion

In Familial Mediterranean fever (FMF), amyloid deposition in any other organ except kidney can also cause morbidity and mortality. Amyloid deposition should be kept in mind in differential diagnosis of FMF patients with refractory cytopenia and bleeding disorder.

## Disclosure of interest

None declared.

